# Directional raids by army ants as an adaption to patchily distributed food: a simulation model

**DOI:** 10.1080/19768354.2018.1497708

**Published:** 2018-07-17

**Authors:** Woncheol Song, Ho-Young Kim, Sang-Im Lee, Piotr G. Jablonski

**Affiliations:** a School of Biological Sciences, Seoul National University, Seoul, South Korea; b Department of Mechanical and Aerospace Engineering, Seoul National University, Seoul, South Korea; c Institute of Advanced Machines and Design, Seoul National University, Seoul, South Korea; d School of Undergraduate Studies, Daegu-Gyeongbuk Institute of Science and Technology, Daegu, South Korea; e Museum and Institute of Zoology, Polish Academy of Sciences, Wilcza, Warsaw, Poland

**Keywords:** Army ant, simulation, raid, foraging

## Abstract

A typical colony of Neotropical army ants (subfamily Ecitoninae) regularly raids a large area around their bivouac by forming a narrow directional column that can reach up to one hundred meters in length. The raid is finished and then relaunched 12–17 times, each time toward different orientation. After completing all bouts the colony relocates to a new area. A hypothetical alternative to this foraging mode is raiding radially and symmetrically by expanding the search front in every direction like a circular bubble. Using an existing agent-based modeling software that simulates army ants’ behavior, we compared the two possible modes of foraging in different food distributions. Regardless of the food patch abundance, the radial raiding was superior to the directional raiding when food patches had low quality, and the directional raiding was favorable when the patches were rich. In terms of energy efficiency, the radial raiding was the better strategy in a wide range of conditions. In contrast, the directional raiding tended to yield more food per coverage area. Based on our model, we suggest that the directional raiding by army ants is an adaptation to the habitats with abundance of high-quality food patches. This conclusion fits well with the ecology of army ants.

## Introduction

The army ants are specialized collective predators that always forage in large groups (Kronauer 2009). Their colony can form a swarm of many thousands of hunters, advancing in a column over a hundred meters long (Couzin and Franks 2003). In the ‘nomadic phase,’ they move their camp every day, but when a colony enters ‘statary phase,’ it launches multiple successive ‘raids (Willson et al. 2011).’ The raids occur about once a day, and they avoid the recently exploited direction (Willson et al. 2011). After depleting a region with about 14–17 raids, the colony relocates to a new area (Willson et al. 2011). Their nomadism depends on their skills to form a ‘bivouac,’ a huge ball of ants that temporarily shelter the young and the queen (Anderson et al. 2002). This set of behaviors has been evolutionarily conserved in more than 200 species of this clade over two continents (Brady 2003).

However, from the exploratory viewpoint, their columnar raid formation is an unusual choice. The search front is narrow (as short as one tenth of the column length; Couzin and Franks 2003), and only the minority of the foragers are exposed to the novel environment. The remaining majority run over the same path as their predecessors did, contributing almost nothing to the search. The successive raids will increase the final coverage area, but still it seems inferior to non-directed search patterns. For example, an *Eciton burchelli* raid can employ 200,000 individuals (Couzin and Franks 2003), and if they were to radiate uniformly from the colony in every direction, they could form an unbroken ring as large as 63,000 body-lengths in diameter. Even with minor workers, this would be nearly 200 meters wide, and it would not miss any single food item within the expanding ‘bubble’. This would provide much better coverage than the column raiding does.

On the other hand, while being inferior in terms of exploration, the directional column raiding enables instant mass transportation after discovery. If the target food source is far away, the colony may save a considerable time by skipping the return trip of the discoverers and the dispatch trip of the recruited transporters. However, to justify having hundreds of thousands of potential transporters following the search front, the colony needs to ensure that the frontiers will find a very rich food patch. Otherwise, it could end up in a waste of time and energy for a very little gain. Therefore, the distribution of food should be a major parameter affecting the advantages of the directional column raiding.

To test the raiding performance under different food distributions, we chose a simulation software (Brown 2008) aimed at modeling *Eciton* species, the popularly studied new world army ants. We modified the program to enable comparison between the naturally occurring ‘directional raiding’ and the hypothetical ‘radial raiding’ strategies. We expected that the radial raiding would provide better coverage, but the directional raiding would yield more food in a certain range of food distributions.

## Methods

We used *AntSpace* 1.1 (Brown 2008), a *NetLogo* (Wilensky 1999) model of army ant raiding behavior. *AntSpace* combines many findings from the past (Deneubourg et al. 1989; Franks et al. 1991; Sole et al. 2000; Couzin and Franks 2003; Brown 2006) and faithfully simulates the army ant behavior. In *AntSpace* 1.1, the ants were assumed to move northward by default, and to stochastically choose their direction by comparing pheromone concentration between the north, the northwest and the northeast grids. We modified the program to accept arbitrary raiding direction and resized the simulated world to 600 × 600 = 360,000 grids. The bivouac was repositioned to the center.

In order to simulate the foraging bout cycle, after 650 simulated time steps all outside foragers were called back to the bivouac. After extra 650 time steps in return phase, the pheromone deposits were all set to 0, and then the raid was re-launched. Fifteen bout cycles were simulated before recording the performance, to imitate *Eciton burchelli* statary phases that launch 14–17 raids before relocating (Willson et al. 2011).

**Figure 1.**
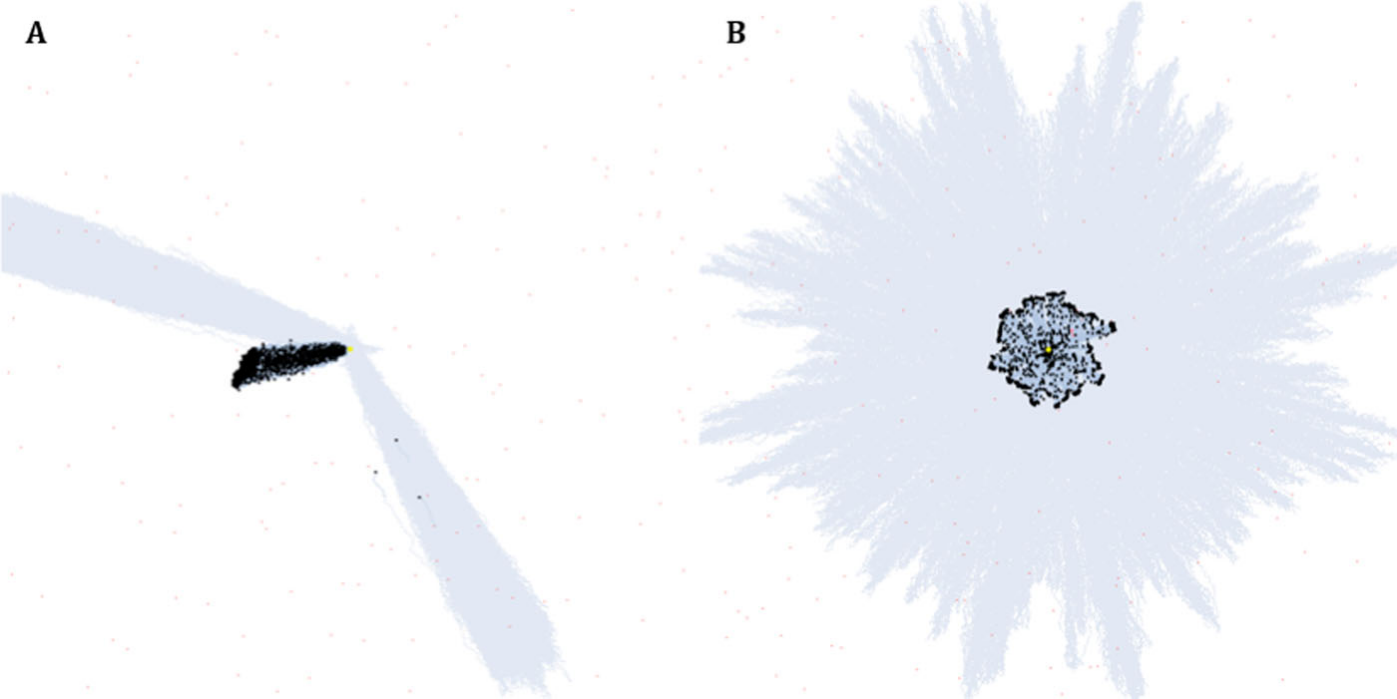
Simulated examples of directional and radial raids. The beginning stages of both directional (a) and radial (b) raids are shown. In both examples, the colony (center) is launching the third raid out of 15 scheduled. The area covered by the previous raids is visualized with pale blue color.

In the directional raid mode, the direction of the new raid was randomly chosen within the half–circle located opposite to the previous raid direction (Figure 1(a)). This was to simulate the actual ant behavior of avoiding the recently raided direction (Franks 1989; Willson et al. 2011). In the radial raid mode, each individual had its own raid direction randomly chosen before leaving the bivouac (Figure 1(b)).

The food patch abundance values were 0.04%, 0.2% and 1%, and the patch quality values were 1, 10 and 100. In *AntSpace* 1.1, the food patch abundance means the proportion of food-loaded grids among all 360,000 grids, and the patch quality is the number of visits required to deplete the grid. This gave 3 × 3 = 9 food distribution conditions. The number of ants were set to 2000, which is similar to the number used in the previous simulations (Sole et al. 2000; Brown 2006). For all the other parameters *AntSpace* requires, we used empirically determined default parameter values provided with the software (Table 1).

**Table 1.** Default parameter values set in *AntSpace* 1.1 (Brown 2008), extracted from the past mathematical models (Deneubourg et al. 1989; Sole et al. 2000) For detailed simulation algorithm which runs on these parameters, please see the publicly available code and information on *AntSpace* 1.1 (Brown 2008). For empirical basis and basic modeling principles underlying the choice of the parameters, please see Sole et al. (2000).

Parameter	Value	Explanation
maxPher-Return	540	threshold pheromone level at which returning ants refuse to deposit additionally
maxPher-Out	51	threshold pheromone level at which outbound ants refuse to deposit additionally
amtPherToRemove	0.005	amount of pheromone evaporated at each time step
amtPherToDrop	47	amount of pheromone deposited by outbound ants at each time step, given that returning ants deposit 10
emptyNodeweightOut	24	basal pheromone level outbound ants perceive from a grid without pheromone
emptyNodeweightIn	24	basal pheromone level returning ants perceive from a grid without pheromone
antsPerStep	10	number of ants that can simultaneously depart from the bivouac per one time step

The ‘coverage area’ of the colony was measured by the count of grids visited by an ant at least once. In order to represent collective energy use by the colony, a variable ‘total movement’ was incremented by 1 every time an ant moved to another grid.

To measure the performance of the colony, the total ‘food collected’ was recorded. It was divided by the total movement or by the coverage area to demonstrate the different aspects of foraging efficiency. Another measurement, the proportion of the collected food to the maximum available amount, represented the ecological impact of the ants on the food resources.

## Results

**Figure 2.**
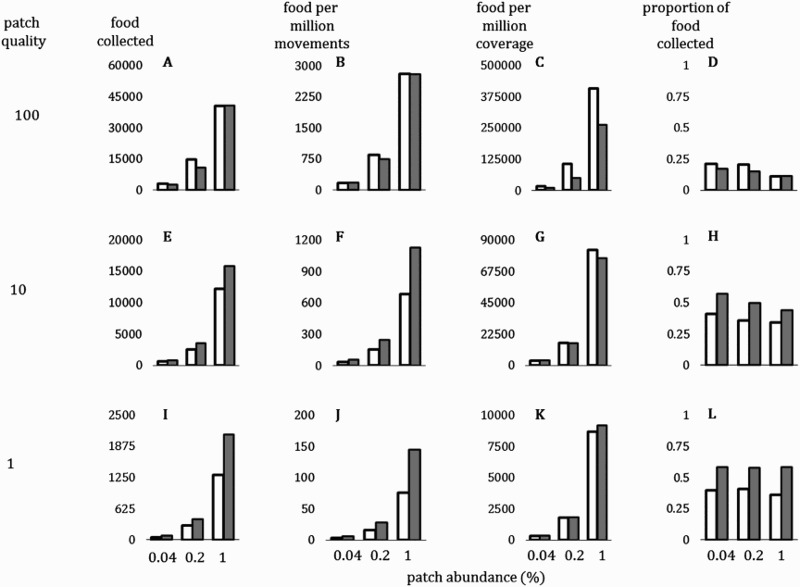
Colony foraging records after 15 raids are completed. White, directional raiding; gray, radial raiding. First column (a, e, i), total number of food obtained. Second column (b, f, j), number of food obtained per one million collective movements. Third column (c, g, k), number of food obtained per one million grids collectively discovered. Fourth column (d, h, l), the collected proportion among the initially available amount. Subpanels are organized in three rows, according to the food patch quality set in the simulation. Each subpanel has horizontal axis for the abundance of food patches.

For low-quality food patches (value 1 or 10) and regardless of the food patch abundance, the radially raiding ants collected 29–63% more food compared to the directionally raiding ants (Figure 2(e,i)). However, for high-quality food patches (100), the directionally raiding ants collected more food overall (Figure 2(a)). A similar general pattern could be seen in the two efficiency measurements (the second and third columns of Figure 2). However, the two measurements differed in details. In terms of the movement efficiency, the food collected in one million collective movements, the radial raiding was the better strategy in general (Figure 2 (b,f,j)). In a wide range of conditions the radial raiding outperformed or closely matched the directional raiding, often winning by margin of almost 90% (Figure 2(f,j)). Only in one condition (the middle plot of Figure 2(b)), the directional raiding was 13% better.

In terms of the coverage efficiency, the food collected in one million explored grids, the directional raiding was a good strategy overall (Figure 2(c,g,k)). For low-quality food patches (i.e. when the patch quality was 1; Figure 2(k)), the directional raiding was less efficient, but the gap was not greater than 11%. For higher-quality patches, the directional raiding performed better, and when the patch quality was 100, the margin was as large as 70–100% (Figure 2(c)).

Food exploitation efficiency (proportion of food collected) was lower in conditions of high-quality food patches, regardless of food patch abundance (Figure 2(d,h,l)). In conditions where radial raiding yielded more food, 34–59% of the total available food was collected (Figure 2(h,l)). On the other hand, in conditions where directional raiding was superior, only 11–21% of the total food was collected (Figure 2(d)).

## Discussion

Although the simulation showed that the directional raiding is generally coverage-efficient, this mode of foraging is not very energy-efficient (the second and third columns of Figure 2). These trends are likely to arise when a large crowd of ants is concentrated in a small number of food patches. In this situation, most of the individuals are active in the already visited area rather than a new unexplored territory, leading to a more thorough search and the higher coverage efficiency. However, the movement efficiency may be negatively impacted by collisions between individuals due to the high density.

Why do the army ants raid directionally? We believe that the coverage efficiency is unlikely to be the ultimate reason, because it is difficult to find a selective pressure that may adaptively constrain the raid coverage. Neotropical army ants are the top predator of the ecosystem (O’Donnell et al. 2007) except when they rarely encounter the anteaters (Willson et al. 2011), and unlike the Afrotropical *Dorylus* (Wilson 1971), inter-colonial conflicts are easily resolved without much mortality (Willson et al. 2011). They also have a set of behaviors specifically tuned to access difficult terrains, such as the ‘living bridge (Reid et al. 2015; Graham et al. 2017)’ or the ‘pothole plug (Powell and Franks 2007),’ implying that they gain benefit by expanding their activity range. Finally, they are nomadic species without permanent shelter, and they frequently relocate to a newer area (Kronauer 2009; Willson et al. 2011; Garnier and Kronauer 2017) suggesting again that they do not pursue smaller coverage.

Can selection in a foraging context explain why the army ants raid directionally? Our model demonstrated that the directional column raiding was not a good foraging strategy to search for scattered small food sources. The model parameters were determined from the observation, so the natural selection could have optimized them for the directional raiding. In contrast, the radial raiding behavior in our simulation did not involve any further optimization to the new foraging regime. Only with the diversification of the initial departing directions, just one simple alteration of the model parameter, the colony gained substantial energetic reward in a wide range of test conditions. Compared to the radial raiding, the 15 directional raids were often inadequate to provide coverage over the full circle of range available to the colony, and left many food patches unexploited.

However, if the food patches were of very high quality, the directional raiding had advantages in various aspects of efficiency. Unlike the radial raiding, the directional raiding could maintain the density of the search front even after a considerably long expedition. This would allow fast and instant concentration of the workforce into a resourceful patch, draining it within a short time. After that, the subsequent raids are unlikely to re-visit the depleted patch. On the other hand, in a radial raiding, it was difficult to recruit the remotely scattered foragers to the discovered patch. The discoverers could lay a pheromone trail back to the bivouac, but the information could not reach the majority of the outside foragers until they come back home. This bottlenecked the transition from exploring to transporting jobs.

The previous research on army ants support the adaptive value of directional raids in habitats with high food patch quality. Army ants, both the neotropical and the Afrotropical groups, are believed to have evolved from a common Gondwanan ancestral clade that preyed on social insect colonies (Berghoff 2003; Brady 2003; Brady et al. 2014), and numerous species still maintain the diet (Berghoff 2003; Ramirez and Cameron 2003; Powell and Clark 2004; Le Breton et al. 2007; Souza and Moura 2008; Kronauer 2009; Powell 2011; Dejean et al. 2014). Others have their diet diversified, but they also generally opt for large preys or rich litter patches (O’Donnell et al. 2005; Kaspari et al. 2011). A study reported that some army ant species generally cherry-pick higher quality patches, only skimming the most convenient 25% of the animal biomass and leave the rest intact (Kaspari et al. 2011). Interestingly, in our simulation, the conditions favorable to the directional raiding were identical to the conditions of less exhaustive exploitations (Figure 2(d,h,l)). To sum up, the directional raiding is a trait closely related to highly rich resources that are not easily exhaustible, both in the real world and in our simulation.

Then, why the majority of other ant species that rely on rich food patches, e.g. the leafcutter and honeydew-harvesting ants, do not utilize the directional column raiding? In these species, a large number of reserve recruits waiting in the nest compensates the downside of the undirected search (Jaffe and Deneubourg 1992). When a recruitment signal is given, the reserves follow the pheromone trail to the newly discovered food source, allowing massive and concentrated exploitation (Jaffe and Deneubourg 1992; Shaffer et al. 2013). Most ant species have highly varied recruitment strategies based on this principle, implying the evolutionary flexibility and universal utility of this behavioral scheme (Hölldobler and Wilson 1990). In contrast, army ant foragers could not benefit from this recruitment scheme, because they leave the bivouac at a much faster rate and save less reserve in the colony (Deneubourg et al. 1989; Sole et al. 2000; Brown 2006). This extreme scout-reserve imbalance is probably for overcoming the specialized prey defense mechanisms (Dejean and Corbara 2014; Dejean et al. 2014; Kessler et al. 2016).

The model does not include some other possible advantages of directional column raiding. First, the large number may allow the ants to overcome physical obstacles collectively by forming self-assembled bridges (Reid et al. 2015; Graham et al. 2017). Second, their large number and density might serve as a protection against predation or competitive aggression. Third, large and mobile prey e.g. living vertebrates could be quickly overwhelmed right after the discovery.

However, these additional benefits from directional raiding by a large number of individuals may not always be high. First, the self-assembled structures are costly commitments of many potentially active foragers, and their traffic enhancement is actually not very great (Powell and Franks 2007; Brunelle 2011). During colony migration, such bridges or rafts would be worth constructing because the queen, pupae, larvae and eggs need to be transported. However, in the foraging context it is difficult to think of a situation where a heavy investment in traffic infrastructure is more important than increasing the coverage area. Second, as noted previously, the selective pressure from predation and competitive aggression is quite low for new world army ants (O'Donnell et al. 2007; Willson et al. 2011). Finally, although some species of army ants do hunt vertebrates (O’Donnell et al. 2005), most army ants primarily feed on social insect colonies (Berghoff 2003). Social insect colonies are immobile food sources and successfully exploitable with non-army-ant behaviors, e.g. by the termite-eating Matabele ants (Villet 1990) and the slave-making social parasites (Alloway 1979; Hasegawa and Yamaguchi 1994). Therefore, we excluded the aforementioned factors from the simulation and focused on the effect of the food distribution only.

In summary, this study illustrates that food distribution alone is sufficient to create ecological situations in which the natural selection may favor column raiding over the radial searching. The future studies should consider variations in different movement parameters as well as in the diet and the colony size to further investigate adaptive value of the raiding behavior.
